# K-Ras prenylation as a potential anticancer target

**DOI:** 10.1007/s10555-020-09902-w

**Published:** 2020-06-10

**Authors:** Marcell Baranyi, László Buday, Balázs Hegedűs

**Affiliations:** 1 2nd Department of Pathology, Semmelweis University, Budapest, Hungary; 2 Institute of Enzymology, Research Centre for Natural Sciences, Hungarian Academy of Sciences, Budapest, Hungary; 3 Department of Thoracic Surgery, Ruhrlandklinik, University Duisburg-Essen, Essen, Germany

**Keywords:** KRAS, Prenylation, Statins, Bisphosphonates, Farnesyl-transferase inhibitor

## Abstract

KRAS is one of the most commonly mutated oncogene and a negative predictive factor for a number of targeted therapies. Therefore, the development of targeting strategies against mutant KRAS is urgently needed. One potential strategy involves disruption of K-Ras membrane localization, which is necessary for its proper function. In this review, we summarize the current data about the importance of membrane-anchorage of K-Ras and provide a critical evaluation of this targeting paradigm focusing mainly on prenylation inhibition. Additionally, we performed a RAS mutation-specific analysis of prenylation-related drug sensitivity data from a publicly available database (https://depmap.org/repurposing/) of three classes of prenylation inhibitors: statins, N-bisphosphonates, and farnesyl-transferase inhibitors. We observed significant differences in sensitivity to N-bisphosphonates and farnesyl-transferase inhibitors depending on KRAS mutational status and tissue of origin. These observations emphasize the importance of factors affecting efficacy of prenylation inhibition, like distinct features of different KRAS mutations, tissue-specific mutational patterns, K-Ras turnover, and changes in regulation of prenylation process. Finally, we enlist the factors that might be responsible for the large discrepancy between the outcomes in preclinical and clinical studies including methodological pitfalls, the incomplete understanding of K-Ras protein turnover, and the variation of KRAS dependency in KRAS mutant tumors.

## Introduction

RAS genes are one of the most frequently mutated oncogenes in several types of cancer and their oncogenic mutations are present up to 25% of all malignancies and mutant RAS is the most common driver oncogene in pancreatic, colorectal, and lung adenocarcinomas [1]. The family of Ras proteins has three classical members, namely N-, H-, and K-Ras. The latter exists in two isoforms, K-Ras4a and K-Ras4b, differing in the hypervariable regions (HVR). All Ras proteins are small GTPases that normally cycle between active GTP-bound and inactive GDP-bound states, tightly regulated by their specific GEF and GAP proteins [2]. Oncogenic mutations occur predominantly on codon G12, G13, and Q61. These mutations impair the ability of GAP proteins to facilitate hydrolyses of GTP to GDP, and therefore the proteins will be constitutively in active, GTP-bound state [1]. Although RAS family members share high similarity, there are differences not only between the hypervariable regions (HVR) of the distinct proteins (166-185 aa) but also in the highly homologous catalytic domain. The catalytic domain can be divided to lobe 1 (1-86 aa) that shares 100% homology among RAS genes, and lobe 2 (87-171) that shows site-specific amino acid variations that probably affects intramolecular dynamics [3].

## Distinct features and non-redundancy of Ras proteins

A number of studies points out to differences and unique features of the distinct RAS members. One of the most obvious observations among these is the differences in mutation patterns. Oncogenic mutations in KRAS are the most common, followed by NRAS and then HRAS. Importantly, KRAS mutations are considered as a negative predictive factor for certain-targeted therapeutic approaches [4–6].

**Fig. 1:**
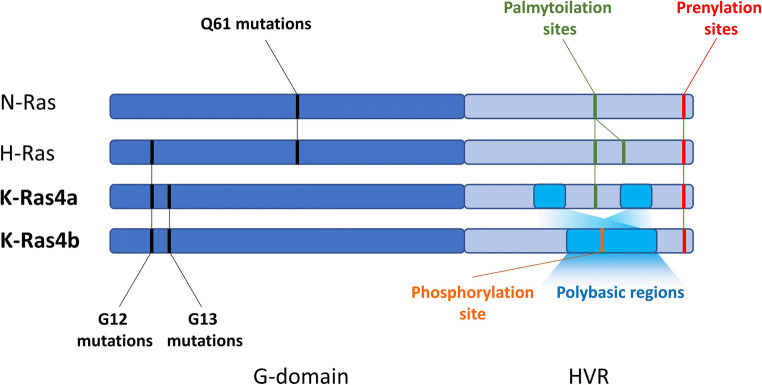
Most common oncogenic mutations and posttranslational modification sites of the HVR regions in the Ras proteins

Of note, mutations of the RAS family show tissue and codon specific bias. For example, KRAS is most commonly mutated in the Ras family in pancreas (60–90%), colorectal (30–50%), and lung (15–30%) adenocarcinomas, while NRAS has the highest mutation rate in melanoma (15–30%) and HRAS in head and neck cancer (6%) [1, 7]. Interestingly, while most of the mutations in KRAS occurs in codon G12 and G13, NRAS is predominantly mutated in Q61 and HRAS shows an intermediate behavior showing similar mutation rates of G12 and Q61(Fig. 1) [1].

Distinct signature of the mutations can be partly explained by differential exposures to carcinogens, as is the case in Q61 NRAS mutations in melanoma linked with UV radiation, or G12C mutations in lung adenocarcinoma in association with smoking. However, non-redundant roles of the different proteins may also be responsible for distinct mutational patterns [1].

Non-redundant roles of the KRAS were demonstrated in several studies. In mice, HRAS inserted to KRAS locus can rescue lethal development deficiencies, but results in cardiomyopathy, suggesting non-redundant functions for KRAS [8]. Interestingly, studies with knockout mice demonstrated that both N-Ras and H-Ras were dispensable for normal development, while mice harboring a homozygous K-Ras null mutation were not viable and die between 12 and 14 days of gestation, with cardiac, liver, neurological, and hematopoietic defects. Based on these findings, only K-Ras is indispensable for embryonic development from the classical *Ras* gene family members [9–12]. In addition, H-Ras was shown to be a more potent activator of Pi3K [13], while K-Ras activates better RAF/MEK/ERK pathway *in vitro* [14]. However, as these experiments were carried out using overexpression methods, results should be interpreted with caution. More recently, genome-editing strategies can generate isogeneic derivatives that harbor distinct mutations of RAS genes expressed from the endogenous loci of the cells. These studies also reveal differences between RAS family members but less dramatic and more context-dependent than the previous ones [15].

One study found that KRAS translation was lower than HRAS in human colorectal cancer cells due to rare codon bias in the former. If they changed KRAS codons to common ones, they observed elevated K-Ras expression. The authors argue that frequent KRAS mutations can be partly explained by low K-Ras protein level, as higher levels should result in oncogene-induced senescence [16]. Somewhat contradictory to this finding, it has been shown that K-Ras expression is significantly higher than the other isoforms in all tissue, although the fact that the experiment was carried out in mice limits the importance of this finding [17]. However, specific features of the amino acid sequence of K-Ras—which are predominantly found in the HVR region—are most expected to regulate its distinct functions.

## Posttranslational modifications of the HVR region

HVR region is the most distinctive and increasingly appreciated feature of Ras proteins. This domain is subjected to a number of posttranslational modifications (PTM) that regulates Ras membrane interactions. In brief, all Ras proteins contains a CAAX motif at the C-termini, responsible for governing prenylation—that is, attaching irreversibly a farnesyl (C15) moiety to the 185 cystein (Fig. 1). Of note, K-Ras protein can undergo alternative geranylgeranylation (C20) in case farnesylation is blocked. Interestingly, this can be observed also in N-Ras, but not in H-Ras [18].

Following prenylation, the AAX motif is cleaved by Rce1, and the carboxyl-termini will be methylated by Icmt [19, 20]. This latter step is considered reversible; however, up to date, no methyl esterase has unequivocally been identified that can remove the C-termini methyl residue in Ras. Nevertheless, polyisoprenylated methylated protein methyl esterase is a potential candidate for this activity [21].

Differences in the HVR region of the KRAS isoforms (Fig. 1) have important consequences in membrane targeting and association. While K-Ras4a—as N- and H-Ras—is subjected to another lipid modification, named palmytoilation, K-Ras4b can only undergo prenylation [22]. However, K-Ras4b contains an isoform-specific polybasic sequence in the HVR, which fosters association to the acidic regions of the plasma membrane (PM) [23]. Of note, phosphorylation of S181 within the polybasic sequence can further modulate K-Ras4b-PM interaction [24].

Though K-Ras4b is considered the more important isoform than K-Ras4a, a recent study demonstrated that K-Ras4a is also widely expressed, and thus can also play an important role in tumor progression. K-Ras4a—similar to N- and H-Ras—can also be targeted to the plasma membrane by regulation of palmytoilation [25]. Palmytoilation happens in the endomembranes—in the Golgi apparatus—and fosters vesicular transport of K-Ras4a to the plasma membrane. Depalmytoilation can occur in the plasma membrane, upon which K-Ras4a is transferred back to the Golgi. Interestingly, K-Ras4a has also two clusters of basic residues that are important—besides palmytoilation—for plasma membrane association and are not found in N- and H-Ras. Genetical modifications of either the polybasic cluster or the palmytoilation site can reduce K-Ras4a membrane association. However, most dramatic inhibition of colony formation and Erk phosphorylation can be observed when the prenylation site is ruined (C186S), suggesting that prenylation is the more important PTM for K-Ras4a signaling [25].

## The importance of KRAS membrane localization

Despite the well-described process of posttranslational modification of K-Ras, it is still debated whether its membrane association is indispensable to exert oncogenic functions especially in light of decades-long failures in targeting K-Ras prenylation.

First, it should be noted that K-Ras proteins are considered to regulate multiple important signaling cascades, showing a highly complex network [26]. We will further focus on the two most-studied signaling pathway regulated by Ras proteins, namely the PI3K/AKT and RAF/MEK/ERK pathway regulating survival and proliferation.

In case of the family of Pi3K, the membrane association is indispensable. Class IA PI3K contains two subunits, the catalytic p110 and the regulatory p85 subunit. The regulatory subunit possesses one SH3 and two SH2 domains. Pi3K phosphorylates phosphoinositol molecules in the plasma membrane (PtdIns4P, PtdIns(4,5)P2) to create PIP3. Pi3K effector proteins (including Akt, Pdk1), through their pleckstrin homology (PH) domains, binds to PIP3, a step that is necessary for them to activate signaling cascades regulating several important cellular processes including cell growth, survival, proliferation, and motility [27]. Of note, to fully activate Pi3K, it seems to be necessary to bind simultaneously to K-Ras and to activated and autophosphorylated receptor tyrosine-kinases (RTKs) at the plasma membrane [28]. Although it is clear that Pi3K should be at the plasma membrane while active but—to the best of our knowledge—no direct experimental data is available whether prenylation deficient K-Ras (e.g., C186S) can activate Pi3K.

Concerning the RAF/MEK/ERK pathway, it is well described that Raf activation needs dimerization [29] which has been shown to happen in the vicinity of the plasma membrane [30]. Raf proteins consist of three closely related genes, namely ARaf, BRaf, and CRaf. Physiologically, upon upstream activation, specific GEFs of the Ras proteins promote exchange of GDP to GTP, thereby activating Ras. GTP-bound Ras recruits Raf through their Ras-binding domain (RBD), fostering their hetero- or homodimerization. Of these, BRaf/CRaf heterodimers are the most potent to activate downstream signaling. Upon dimerization, phosphorylation of the Raf proteins occurs; however, mechanistically, it is unclear whether it happens by auto or transphosphorylation. Following phosphorylation, the Raf dimers in turn phosphorylate Mek, passing on the signal to downstream members of the signaling cascade [31]. Of note, several studies indicated that K-Ras localization is not homogenous at the plasma membrane, rather it appears to signal through nanoclusters, small groups of Ras proteins at distinct compartments part of the plasma membrane [32]. It has also been shown experimentally that K-Ras can form homodimers [33]. One rationale for the potential role of Ras dimerization is based on the dimerization-dependent nature of Raf activation and a 1:1 stochiometric ratio of Ras-Raf interaction domains of the given proteins (i.e., the effector domain in Ras- and Ras-binding domain of Raf). Moreover, blocking K-Ras dimerization and nanoclustering—either by genetic means or with specific antibodies—inhibits the efficacy of BRaf/CRaf heterodimerization and MAP kinase (MAPK) activation [33, 34]. It has also been shown that prenylation is necessary for Ras dimerization-dependent Mapk activation. An FKB-derived dimerization domain (DD) was genetically fused to the N termini of PAmCherry1-K-Ras^G12D^. DDs can be “crosslinked” with a dimerizing small molecule (that can bind two and only two DD). Upon addition of dimerizing agent, they observed K-Ras dimerization by photoactivated localization microscopy and increased Mapk activation by western blot analyses. However, when DD sequence was fused to a prenylation deficient C186S KRAS, addition of dimerization agent failed to enhance K-Ras dimerization and no activation of Mapk could be observed [35]. The proposed K-Ras dimerization interface is between α-helix4 and α-helix5 of the opposing monomer. The dimer is stabilized by a salt-bridge of D154 of one with R161 of the other K-Ras protein, as charge reversal D154Q mutation efficiently abrogated dimerization. However, protein-protein interaction mediated by α-helix4 and α-helix5 interface are suggested to be a relatively weak; thus; it seems reasonable that plasma membrane anchoring is needed to facilitate higher local concentration of K-Ras and bring them in close proximity so that dimerization can occur [33]. Moreover, Raf cysteine-rich domains (CRD) have been shown to promote Raf membrane association which in concert with Ras binding, stabilizes the signaling complex at the PM, and probably facilitate signaling by reducing the Ras-Raf-binding fluctuations [36]. Altogether, we can conclude that experimental data suggest that Raf activation requires Ras membrane localization, nanoclustering, and/or dimerization.

## HVR-associated-specific features of KRAS

All Ras proteins are tightly regulated by guanin-nucleotide exchange factors (GEFs) and GTPase-activating proteins (GAPs) that is necessary for their proper functions. Upon upstream stimuli, GEFs activates Ras proteins by promoting GDP to GTP exchange, which results in conformational changes. As intrinsic GTPase activity of Ras proteins are relatively slow, their inactivation needs GAPs that foster their GTP hydrolysis rates [37]. Activating mutations prevents this inactivating mechanism, resulting in elevated Ras signaling.

However, K-Ras is differentially affected by several unique regulators of its activity compared to other Ras family members. It selectively interacts with calmodulin, protein kinase c (PKC), and in case of K-Ras4b isoform, with phosphodiesterase 6 delta (Pde6δ). Of note, these interactions are all mediated through K-Ras HVR.

It has been shown that PKC can phosphorylate K-Ras4B on S181, reducing the strong positive charge of the HVR’s polybasic sequence, thereby weakening interaction with the negatively charged PM, potentially fostering membrane dissociation [24]. Interestingly, Ca2+-binding calmodulin was found to bind K-Ras through its farnesylated C-termini in a hydrophobic pocket [38]. Of note, this interaction can hinder PKC-mediated phosphorylation of S181, thereby preventing an important negative regulatory mechanism. Furthermore, it has been suggested that calmodulin along with K-Ras and Pi3K can form a ternary complex, possibly facilitating K-Ras-mediated activation of Pi3K—even in the abscence of upstream RTK signaling [39]. However, this suggestion has yet to be proved experimentally. Of note, in the absence of K-Ras farnesylation, calmodulin could probably not bind K-Ras, thereby altering Pi3K activation.

Furthermore, in case of K-Ras4b, but not K-Ras4A, it has been shown that Pde6δ chaperon protein mediates PM targeting of K-Ras4b [25]. Pde6δ—like calmodulin—also binds K-Ras4B through the farnesylated C-termini sequestering the hydrophobic protein in the cytosol. By computational modeling and experimental data, Schmick and colleagues demonstrated that—Pde6δ can extract K-Ras4b from the membranes. ADP-ribosylation factor-like protein 2 (Arl2) interacting with Pde6δ can foster release of farnesylated K-Ras, facilitating its accumulation at the perinucleolar membranes. This leads to K-Ras4b being anchored to the recycling endosomes, which will transfer K-Ras4b to the PM. This process is important because the endomembranes—given their relatively higher surface—would compete for K-Ras4b with the PM; thus, it would not be specifically enriched in the PM [40].

## Classes of known prenylation inhibitors

**Fig. 2:**
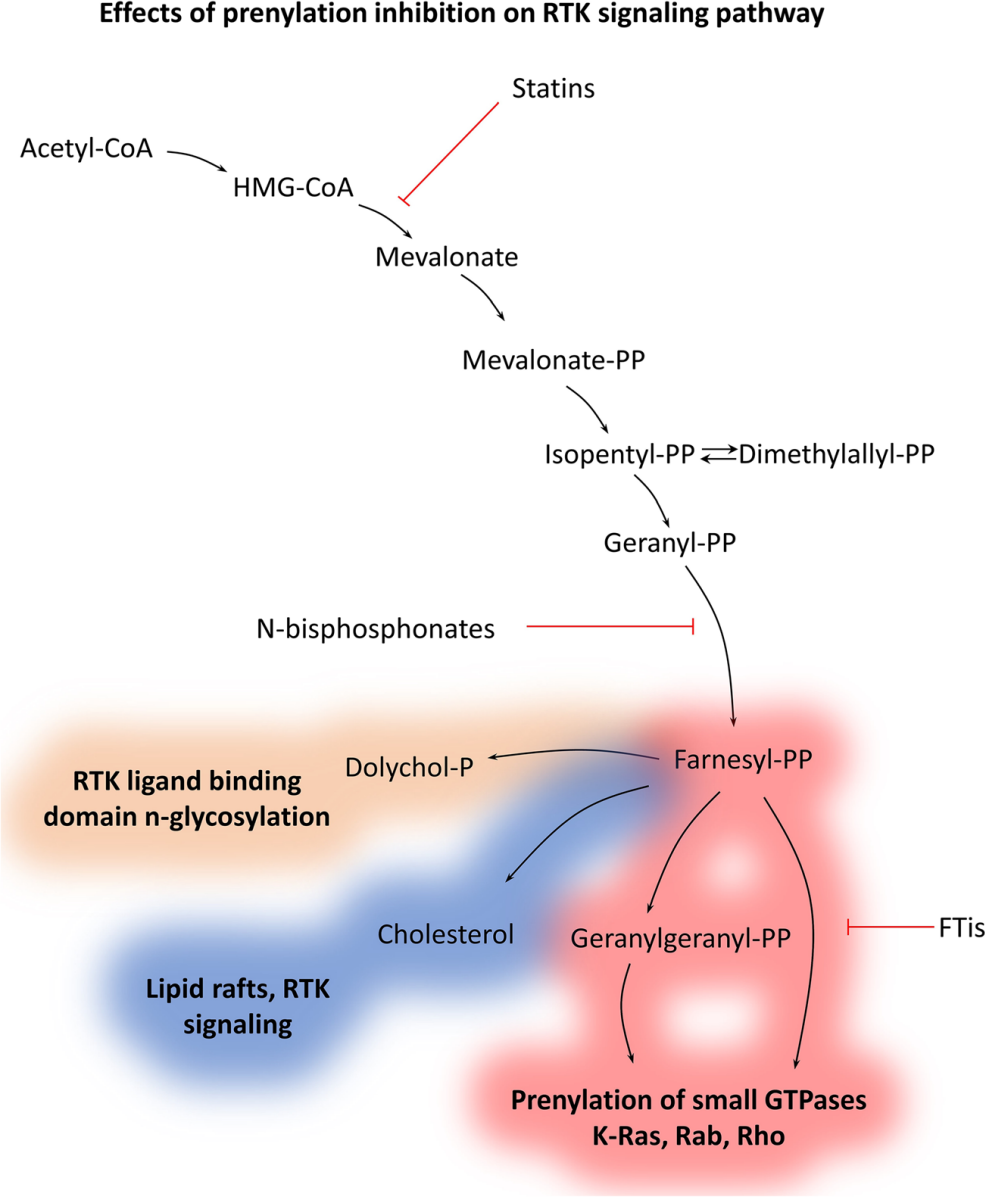
Effect of prenylation inhibition on receptor tyrosine kinase (RTK) pathway. Distinct classes of prenylation inhibitors act on different levels of the mevalonate pathway. Statins and N-bisphosphonates shut down farnesyl-PP synthesis, leading to—besides inhibition of prenylation—depletion of dolichols and cholesterol. Dolichols are involved in N-glycolysation that is essential for proper ligand binding of certain RTKs, like EGFR. Cholesterol is a major compound of lipid rafts, specific microdomains of the plasma membrane functioning as signalization hubs in many major signaling pathways (e.g., EGFR and HER2). Prenylation inhibition concerns many major cellular process, like proliferation, survival, migration (K-Ras, Rheb Rho), vesicular transport, and autophagy (Rab). Interference with this metabolic pathway likely leads to pleiotropic effects

Conventionally, prenylation inhibition of K-Ras has been associated primarily with the introduction and failure of farnesyl-transferase inhibitors (FTis). However, there are additional two clinically approved class of drugs that are considered as potent prenylation inhibitors, namely statins and nitrogen-containing bisphosphonates (N-bisphosphonates) [41]. Below, we will discuss these distinct drug classes alongside with FTis that are mechanistically differ from statins and N-bisphosphonates. Of note, FTis are only in clinical phase II trials for HRAS mutant head and neck cancers (clinicaltrials.gov ID: NCT03496766; NCT02383927; NCT03719690) and are not clinically approved for other diseases in contrast to statins and N-bisphosphonates. These drugs interfere with distinct steps of the prenylation process which are shown in Fig. 2.

Mechanistically, both statins and N-bisphosphonates are metabolic inhibitors that prevent synthesis of prenylation substrates. However, they act on different levels of the mevalonate pathway. One documented mechanism of action that can be different between statins and N-bisphosphonates is that the latter—by inhibiting IPP conversion to farnesyl-PP—facilitates accumulation of IPP that in turn will be conjugated to AMP resulting in cytotoxic ATP analogue ApppI [42]. However, other mechanisms like the dynamics of inhibition of the mevalonate pathway may also lead to differential effects of statins and bisphosphonates.

Statins inhibit the HMGCoA-reductase enzyme that converts HMGCoA to mevalonate. Of note, this action is considered as the rate-limiting step in this metabolic pathway. In the absence of mevalonate, the biosynthesis of upstream products is shut down. Depletion of mevalonate has been demonstrated as main mechanism of action of statins, as addition of exogenous mevalonate diminishes inhibitory effects of the drugs [43]. Of note, though statins are being used for hypercholesteremia, there are some hurdles that can prevent their effectiveness *in vivo.* First, dose-limiting toxicities may impede reaching effective plasma concentration of these drugs [43]. Second, statins may interact with other drugs that can also limit their use for combination therapies [44].

Nitrogen-containing bisphosphonates—but not other bisphosphonates—have been shown to inhibit farnesyl-pyrophosphate synthase and—to some extent—geranylgeranyl pyrophosphate synthase. This also results in the blocking of upstream product synthesis similar to statins and addition of farnesyl-OH and geranylgeranyl-OH (both then converted in the cells to farnesyl-PP and geranylgeranyl-PP, respectively) hinders the inhibitory effects of N-bisphosphonates [45]. Interestingly, intermediates between mevalonate and farnesyl-PP are not involved in specific cellular processes, and thus statins and N-bisphosphonates are expected to have similar inhibitory profiles. Non-nitrogenous bisphosphonates differ from N-bisphosphonates in mechanism of action, as they exert their anticancer effects through formation of non-hydrolysable cytotoxic ATP analogues [46]. All bisphosphonates have high affinity to mineral material of the bones limiting their use to bone-related diseases like osteoporosis or bone metastases [47]. However, lipophilic N-bisphosphonates have been developed that can overcome this hurdle widening therapeutic applications for non-bone-related conditions [48]. In addition, bisphosphonates have favorable side effect profiles as the main documented complication is osteonecrosis of the jaw [49], which probably would occur to a lesser extent using lipophilic bisphosphonates. However, it has to be noted that healthy renal function with a glomerulus filtration rate > 35 ml/min is also necessary for safe bisphosphonate treatment [50].

In contrast, mechanism of action of FTis is markedly different. They specifically block activity of the farnesyl-transferase enzyme resulting in a narrower range of inhibition than the other two above described class of drugs inhibiting only farnesylation. Historically, FTIs were expected to block Ras signaling but led to an often-cited failure that hindered research targeting Ras prenylation for years (Table 1). It was concluded that the failure was due to alternative geranylgeranylation of K and N-Ras [41, 51]. However, ongoing trials will show whether mutant H-Ras—that can only be farnesylated—can still be a valid target of FTis in the future (clinicaltrials.gov ID: NCT03496766; NCT02383927; NCT03719690).

**Table 1:** Phase II–III trials of farnesyl-transferase inhibitors in pancreatic, colorectal, and non-small cell lung cancer (upper table) and phase II trials utilizing statins for KRAS mutant solid tumors (lower table). Only trials with published results are listed

ID	Phase	Drug	Tumor type	Published results
PMID: 15459217	Phase III	Tipifarnib monotherapy	Refractory advanced colorectal cancer	Well tolerated, no improved OS compared to best supportive care
PMID: 16683076	Phase II	Tipifarnib monotherapy	Advanced colorectal cancer	Concluded to be not effective as monotherapy
clinicaltrials.gov ID: NCT00005648	Phase III	Gemcitabine with or without tipifarnib	Advanced pancreatic cancer	Tolerable toxicity, no improved OS compared to gemcitabine + placebo
PMID: 12663718	Phase II	Tipifarnib monotherapy	Metastatic pancreatic cancer	No antitumor activity
PMID: 12721252	Phase II	Tipifarnib	Advanced non–small-cell lung cancer	Minimal clinical activity, no improved OS
PMID: 16028213	Phase II	Lonafarnib with paclitaxel	Non-small cell lung carcinoma	Clinical benefit in 48% of patients, minimal toxicity
PMID: 12176785	Phase II	Lonafarnib	Metastatic colorectal cancer refractory to 5-fluorouracil and irinotecan	No objective response, gastrointestinal toxicities
PMID: 26386973	Phase II	Cetuximab plus simvastatin	Previously treated KRAS mutant metastatic colorectal cancer	22% free of progression until primary endpoint, treatment was concluded to be not effective
PMID: 26053280	Phase II	Pamitumumab plus simvastatin	Previously treated KRAS mutant metastatic colorectal cancer	7% free of progression until primary endpoint, treatment was concluded to be not effective
PMID: 24468885	Phase II	Cetuximab/irinotecan plus simvastatin	Previously treated KRAS mutant colorectal cancer	Observed clinical activity and tolerability, response claimed to be associated with RAS signature
PMID: 24162380	Phase II	Gemcitabine with or without simvastatin	Advanced pancreatic cancer	No increased toxicity, no clinical benefit observed

## *In vitro* sensitivity of KRAS mutated tumor cells to prenylation inhibitors

There are several reviews about the *in vitro* effects and mechanism of the different classes of prenylation inhibitors like statins, bisphosphonates, and FTis [41, 43]. Below, we are focusing on the predictive potential of KRAS mutations and the efficacy of the aforementioned classes of drugs.

**Fig. 3:**
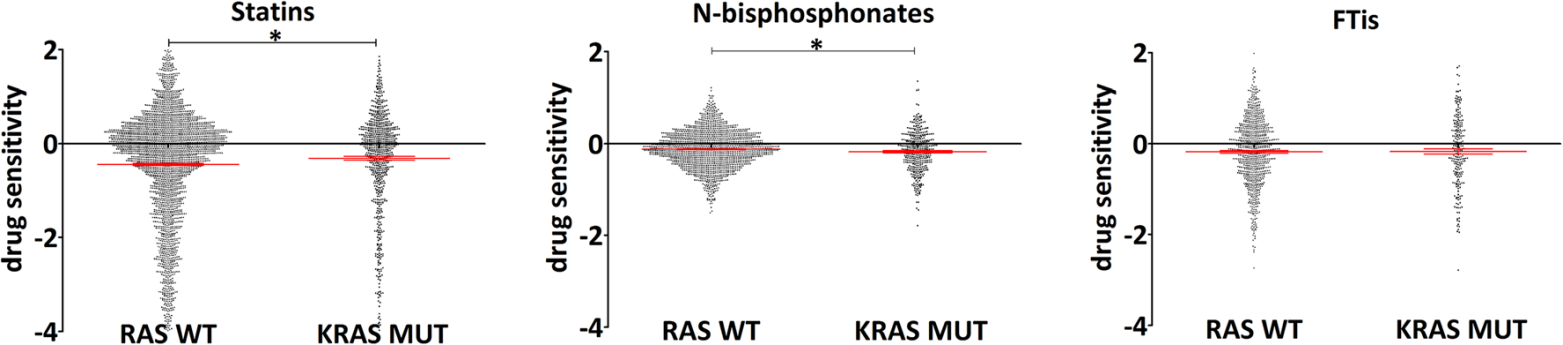
Drug sensitivity values from PRISM repurposing primary screen of all cell lines independent of tissue of origins. Efficacy of statins, nitrogen-containing bisphosphonates, and farnesyl-transferase inhibitors on KRAS mutant cells versus RAS wild-type cell lines are showed by analyzing the open access data from [52]. Drugs included for the analyses are listed in Table 2. KRAS mutant cell lines were more resistant to statins *(*RAS WT mean − 0.123, SEM 0.024; KRAS MUT mean – 0.176, SEM 0.040), while the opposite can be observed in response to N-bisphosphonate treatment (RAS WT mean – 0.124, SEM 0.010; KRAS MUT mean − 0.176, SEM 0.022). Statistical significance was established in *p* < 0.05, using two-tailed *t* test. Number of cell lines included in the analyses RAS WT *n* ~ 390; KRAS MUT *n* ~ 103 (there is a small variation between the numbers of cell lines with available data for the distinct drugs)

**Fig. 4:**
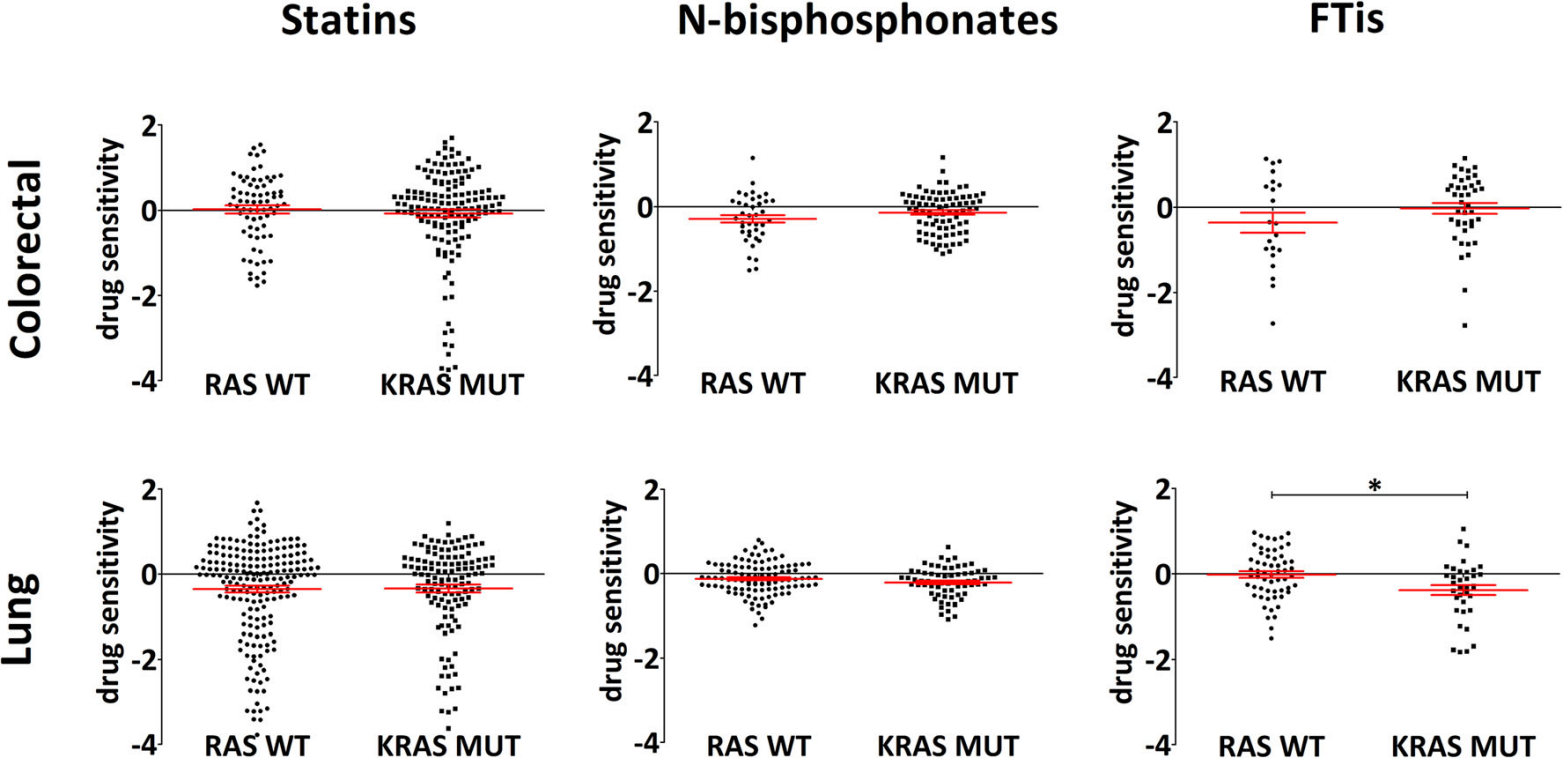
Drug sensitivity values from PRISM repurposing primary screen of lung and colorectal cancer cells. Efficacy of statins, nitrogene-containing bisphosphonates, and farnesyl-transferase inhibitors on KRAS mutant cells versus RAS wild-type cell lines are showed. List of drugs included for the analyses are listed in Table 2. Interestingly, statistically significant difference could only be observed in lung cancer cell lines treated with FTis (RAS WT mean – 0.021, SEM 0.076; KRAS MUT mean – 0.385, SEM 0.117). Note the opposite trends in sensitivity between N-bisphosphonate- and FTi-treated colorectal and lung cancer cells. Statistical significance was established in *p* < 0.05, using two-tailed *t* test. *n* analysis was performed using the open access data of [52]. Number of cell lines included in the analyses for colorectal cancer cell lines: RAS WT *n* ~ 10; KRAS MUT *n* ~ 20; for lung adenocarcinomas: RAS WT *n* ~ 28; KRAS MUT *n* ~ 18. There is a small variation between the number of cell lines with available data for the distinct drugs

**Fig. 5:**
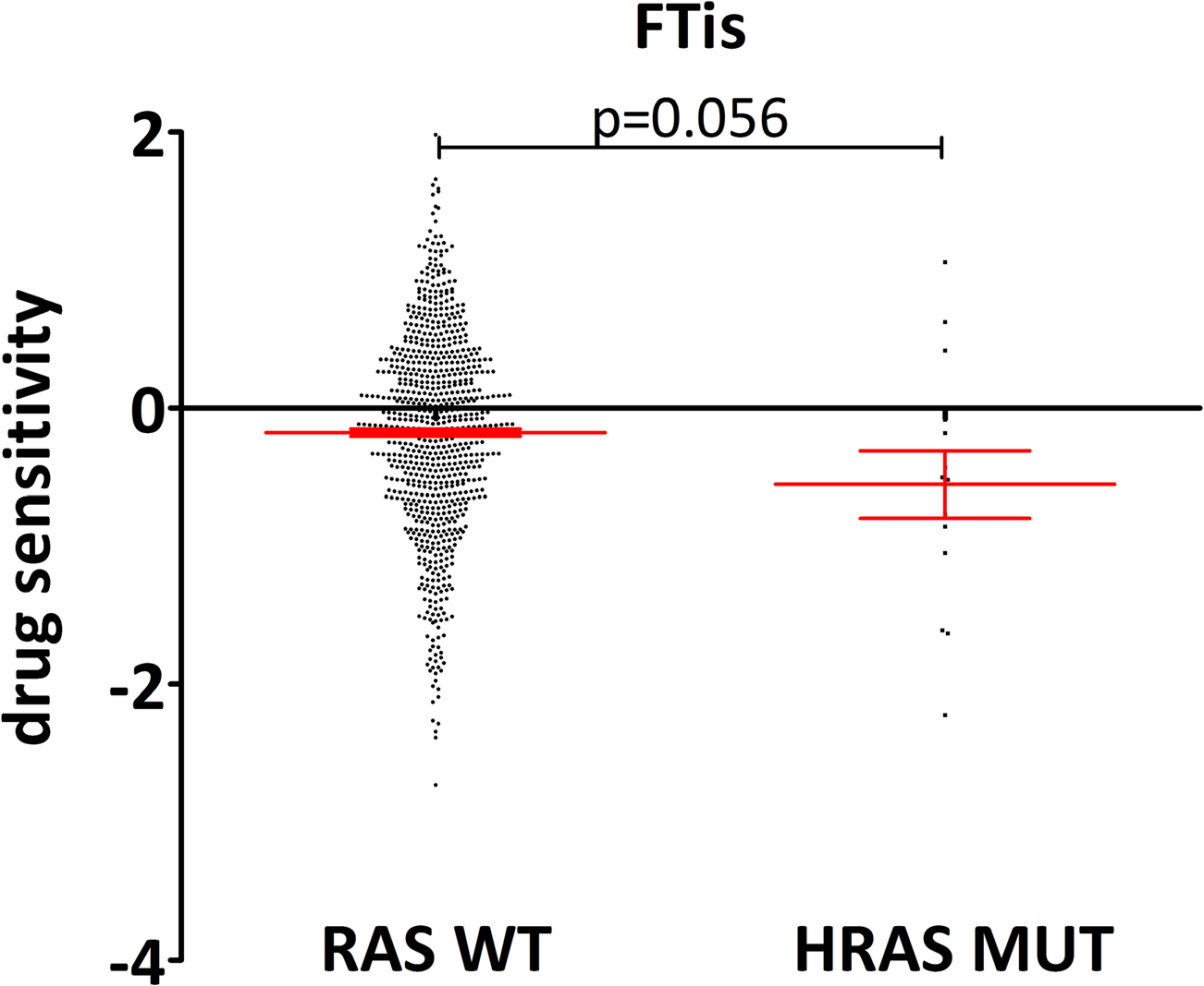
Drug sensitivity values from PRISM repurposing primary screen of lung and colorectal cancer cells. Efficacy of farnesyl-transferase inhibitors (tipifarnib and lonafarnib) on HRAS mutant cells versus RAS wild-type cell lines is presented. A strong tendency was observed, HRAS mutant cells were more sensitive to FTi treatment than RAS wild-type cells (RAS WT mean – 0.178, SEM 0.026; HRAS MUT mean – 0.554, SEM 0.243). *p* value was calculated using two-tailed *t* test. The open access data of [52] was used for the analysis. Number of cell lines included in the analyses: RAS WT *n* = 396; HRAS MUT *n* = 7 (created by Graphpad Prism 5 software)

Since the majority of the aforementioned studies only characterized a limited number of cell lines, we performed an analysis of the publicly available DEPMAP database (https://depmap.org/repurposing/) to provide direct insight for differences between drug classes, tissue-specific effects using drug sensitivity data from the Repurposing Primary Screen [52]. The screen utilizes 5-day-long treatments of 750 barcoded cell lines presenting sensitivity values of numerous drugs at 2.5 μM concentration. We used data only from the primary screen because the secondary screen does not contain results for all three classes of prenylation inhibitors. Data provided here show sensitivities of three classes of prenylation inhibitors—statins, N-bisphosphonates and farnesyl-transferase inhibitors—comparing K-Ras hotspot mutant cell lines (G12, G13, and Q61 mutations based on COSMIC and TCGA database) to Ras wild-type cell lines. In order to lower drug-to-drug differences, we treated all available drugs (of PRISM repurposing primary screen) of the distinct classes together. List of drugs included is in Table 2. Sensitivity data of the corresponding drugs combined with RAS mutational status and lineages using the https://depmap.org/portal/interactive/ tool. Results were downloaded, manually reviewed, and combined according to drug classes (Table 2) and RAS mutational status. Of note, for tissue-specific analyses (Fig. 4), all colorectal cancer cell lines were included; however, in case of lung cancer only, data of lung adenocarcinoma cells were used. Respective numbers of the cell lines included in the corresponding analyses are indicated in the description of Figs. 3, 4, and 5.

**Table 2:** List of drugs of the distinctive classes of prenylation inhibitors involved in the analyses showed in Figs. 3, 4, and 5

Statins	N-bisphosphonates	FTis
Atorvastatin	Alendronate	Lonafarnib
Lovastatin	Pamidronate	Tipifarnib
Mevastatin	Ibandronate	
Pitavastatin	Neridronate	
Pravastatin		
Rosuvastatin		
Simvastatin

Interestingly, if we compared sensitivity of all available cell lines in the PRISM primary screen, we found marked differences between the three classes of drugs (Fig. 3). Of note, statins and bisphosphonate, expected to behave similarly, apparently showed opposite effects on K-Ras mutant versus Ras wild-type cells. K-Ras mutants were more resistant to statins while exhibited significantly higher sensitivity to bisphosphonates in the same comparison. These significant differences demonstrate that indeed these classes of drugs have distinct impact on K-Ras mutant cells. Interestingly, no difference could be observed in sensitivity towards FTis between K-Ras mutant and wild-type cells.

An additional important aspect is that the tissue of origin might have an impact on the biology and drug sensitivity of KRAS mutant cells [53]. It has been shown that prognostic value of KRAS mutations can be dependent on the site of metastasis, emphasizing context-dependent-specific biological features of mutant KRAS [54–56]. In addition, mutational patterns may also modulate tissue-specific differences. For example, KRAS G12C, G12V, G13D, or Q61L exhibit differential intrinsic GTPase activity, sensitivity to GAP-mediated inactivation, or activation by GEF proteins [57, 58]. Thus, distinct properties of the different mutations along with tissue-specific mutational patterns may mediate differential sensitivity to prenylation inhibitors.

Accordingly, when we compare the efficacy of these three classes of these prenylation inhibitory drugs depending on tissue of origin, pronounced sensitivity differences emerged. Figure 4 shows the comparisons of lung and colorectal adenocarcinomas. Significant differences can only be found in lung adenocarcinoma; however, distinct trends can be observed depending on tissue of origin. For example, KRAS mutant colorectal cell lines show higher resistance against N-bisphosphonates (*p* = 0.12) and FTis (*p* = 0.17) when compared to wild-type cells. Interestingly, we can see the opposite effects on lung adenocarcinomas; KRAS mutant lung adenocarcinoma cell lines are more sensitive to N-bisphosphonates (*p* = 0.136) and to FTis (p = 0.007) than wild-type cells. There are no statistically significant differences when analyzing statins (*p* > 0.5).

These results clearly demonstrate the tissue-specific effects of prenylation inhibition on K-Ras mutant cells. Moreover, this analysis points to new questions, for instance, why FTis can inhibit K-Ras mutant lung cancer cells despite the well-characterized alternative geranylgeranylation of K-Ras that is supposed to be a potential resistance mechanism against this class of prenylation inhibitors.

Additionally, since H-Ras cannot be geranylgeranylated, we investigated how FTis affect H-Ras mutant cell lines (Fig. 5). Albeit there are only a limited number of HRAS mutant cell lines, we found that FTis tended to have higher inhibitory effects on them when compared to RAS wild-type cell lines (*p* = 0.056). Of note, there are ongoing trials investigate currently FTi efficacy in HRAS mutant cancer (clinicaltrials.gov ID: NCT03496766; NCT02383927; NCT03719690)). These trials will clarify whether at least in HRAS mutant tumors farnesylation inhibition is a clinically effective approach.

Furthermore, differences in isoform expression can also influence sensitivity to prenylation inhibitors. Of note, K-Ras4b is known to have far the highest affinity towards farnesyl-transferase enzymes, followed by K-Ras4a [59]. It has been recently described that K-Ras4a is widely expressed in different types of cancers, indicating that differential expression can substantially influence KRAS signaling and regulation [25]. In addition, it has been shown that prenylation of small GTPases is regulated by different isoforms of rap1 GTPase-GDP dissociation stimulator 1 (RAP1GDS1 or alternatively SMGGDS) gene. Elevated expression of Smggds has been associated with tumor progression in breast cancer and lung adenocarcinoma [60, 61]. Interestingly, it has been shown that expression changes which results in an aberrant ratio of Smggds 607:558 isoforms alters protein prenylation, and thereby regulates their activation. This can be explained by different functions of the isoform 607 and isoform 558: while the former interacts with the preprenylated, newly synthetized small GTPases, the latter interacts with the prenylated small GTPases. [62]. We suggest that tissue-specific expression differences of Smggds can alter sensitivities towards prenylation inhibition.

Of note, as prenylation is irreversible, prenylation inhibitors can only interfere with newly synthetized K-Ras proteins. However, despite decades-long research on K-Ras proteins, little is known about K-Ras degradation rates and turnover. Many mechanism has been proposed to regulate K-Ras degradation, including Lztr1-cul3 [63], Nedd41 [64], and β-Trcp1 [65]. WNT/β-CATENIN signaling has been shown to regulate Ras stability. The authors argue that Gsk3b-mediated phosphorylation of Ras T144 and T148 induces proteosomal degradation of Ras proteins. However, β-catenin—through binding to Ras—can hinder phosphorylation by GSK, thereby inducing its stability. Stabilization of β-catenin—that can occur either by BCAT1 mutation or by loss of Apc, Axin, etc—would thereby lead to increased stability of K-Ras [66]. Mutations in the WNT pathway frequently occur in colorectal cancer and have been shown to cooperate with KRAS signaling [67]. Resistance to prenylation inhibitors of KRAS mutant colorectal cancer (Fig. 4) may be partly due to increased stability of K-Ras proteins mediated by mutations in WNT signaling pathway.

## Resistance of mutant K-Ras proteins against prenylation inihibition

Altogether, experimental data provides strong evidence that posttranslational modification and specifically prenylation of K-Ras are important to exert its functions. For decades, this approach was considered the only option against the undruggable-mutated K-Ras protein; however, despite all promising experimental data, none of the clinical trials were successful Table 1. Accordingly, we enlist the potential resistance mechanisms that lead to the unsuccessful clinical translation.

At least for FTis, lack of clinical activity on mutant K-Ras harboring cases can be explained by alternative geranylgeranylation that rescue membrane association of the oncoprotein [41]. However, statins and bisphosphonates blocking the whole mevalonate pathway shows currently no clear escape route for K-Ras. It seems contradictory to compare excess literature arguing that statins and bisphosphonates interfere with the Ras signaling pathway with the notion that no specific and direct activity has been reported for these drugs on mutant K-Ras in the clinics.

Unfortunately, it has to be pointed out that common methodological errors are also responsible for this discrepancy. For example, it was recently showed that many of the commercially available Ras antibodies do not recognize specifically their intended target and/or show affinity to other proteins that could lead to false results and conclusions [68]. Furthermore, none of the most frequently used antibodies except for SC-31 (from Santa Cruz Biotechnology) for N-Ras performed reliable results in immunocitochemistry; however, many studies show immunocitochemistry-based proof for prenylation-induced mislocalization of Ras proteins.

Western blot experiments performed without strictly validated antibodies can also show false results even if molecular weight of the given protein bands is similar to those of Ras proteins. M, RRas, and many other small G proteins share high homology with KRAS and the other genes, and will appear exactly at the same height, and also undergo prenylation. Only rigorous validation of the given antibody can exclude the possibility that changes—either in prenylation or activation status (e.g., via Ras-pull down assay)—is due to other proteins very similar to K-Ras, N-Ras, or H-Ras.

Another common pitfall is to conclude successful Ras inhibition merely based on changes of Erk phosphorylation. Although one of the most important signaling pathway regulated by Ras proteins is indeed the Raf/Mek/Erk pathway, changes in Erk activation status not necessarily mirrors inhibition of Ras. PI3K-Erk crosstalk [69], impaired upstream signals or regulatory feedback loop activation [70] can greatly affect phosphorylation of Erk; thus, it is always necessary to perform other validation experiments for mechanistic conclusions. For instance, we have recently demonstrated that N-bisphosphonate treatment resulted in elevated Erk phosphorylation in K-Ras mutant harboring colorectal cancer cells, and this effect diminished upon knockout of the mutant allele [71]. Furthermore, N-bisphosphonates can also modulate tumor microenvironment, for example, zoledronic acid has been shown to activate γδT cells [72], or modulate tumor xenograft vascularization [73]. These results show that we are still far away from fully understanding how prenylation inhibition affects Ras signaling and tumor progression. With the development of better reagents and by avoiding these methodological pitfalls, we will get a clearer picture on how prenylation inhibitors actually affect Ras signaling.

Besides these technical issues, the promiscuous nature of metabolic inhibitors of the mevalonate pathway further complicates the K-Ras prenylation studies. Approximately 2% of all translating proteins get prenylated; many of these are playing indispensable roles in many cellular process [74]. These includes many small GTPases, like Rheb regulating the PI3K/AKT/mTOR pathway; Cdc42 controlling cell cycle [75]; Rho and Rac involved in regulation of cell skeleton and motility [76]; Rab family proteins that controls intracellular vesicular transport, endo and exocytosis, and autophagy [77]. For example, our group has recently demonstrated that the efficacy of prenylation inhibition was associated with reduced Rheb prenylation [78] or cells showed sensitivity depending not only on NRAS but also on BRAF/PTEN mutational status [79]. In addition, in several studies, effects of statins or bisphosphonates are mainly mediated by inhibition of RhoA/B prenylation [41, 43].

These results suggest that mutant KRAS-dependent effects of prenylation inhibitors can be hindered by other small GTPases that are more sensitive or more dependent on prenylation. Emphasizing the irreversible nature of prenylation, we would like to point out again that prenylation inhibitors only affect newly synthetized proteins. Therefore, protein turnover seems to be a major factor determining sensitivity towards prenylation inhibition. Unfortunately, no reliable data are currently available on K-Ras protein turnover except for some sporadic reports that not allow exact determination of dynamics of degradation and synthesis rates [65, 80]. However, even these uncomplete results show that K-Ras half-life is likely more than 12 or even 24 h, alongside with assumptions based on the “*n*-rule” suggesting K-Ras protein stability, as the second amino acid following the cleaved initiator methionine is threonine that is considered a “stabilizing” residue [81]. Higher stability of K-Ras compared to other GTPases may be credited for lack of K-Ras mutation dependence of prenylation inhibitors and probably can be the major obstacle for evaluation of drug effects on K-Ras. Approaches that are more specific to K-Ras prenylation—like inhibition of C-terminal binding by Pde6δ [82] —would counteract the obstacles derived from the (preassumed) low turnover rate of K-Ras. Nevertheless, critical investigation of K-Ras degradation and synthesis dynamics are urgently needed.

In addition, not all KRAS mutant tumors exhibit similar RAS dependence. It has been shown that proliferation and growth of a significant proportion of KRAS mutant cancer cell lines are not dependent on mutant KRAS. Either pharmacologic inhibition of KRAS G12C [83] or genetic ablation by CRISPR-Cas9 [84] revealed a subset of cell lines that exhibits *de novo* resistance to depletion of KRAS function. Interestingly, PI3K inhibition along with KRAS targeting exhibited pronounced anticancer effects [83, 84]. Of note, PI3K pathway are a potential candidate not only in KRAS mutant cancers, but also in solid tumors harboring NRAS mutations [85].

Furthermore, consequences of mevalonate pathway shutdown do not end in inhibition of prenylated proteins. The whole squalene biosynthesis is based on this metabolic pathway including synthesis of cholesterol, steroid hormones like estrogens, and dolichols (involved in protein glycosylation) [86]. This means (at the intracellular level) that signaling from the plasma membrane will be drastically changed as cholesterol; an important component of the lipid rafts will be depleted (Fig. 2). Lipid rafts are specific microdomains within the membranes that are considered as important hubs for different signalization pathways [87].

Of note, lipid raft localization of EGFR has been shown under physiological conditions [88]. Interestingly, inhibition of Her2 receptor localization to lipid rafts blocked breast cancer cell proliferation [89], and depletion of cholesterol by lovastatin treatment diminished resistance of breast cancer cells to gefitinib treatment [90]. Furthermore, mevalonate pathway shutdown blocks synthesis of dolichols that are implicated in N-glycosylation of proteins. Of note, EGFR is known to be heavily glycosylated and this process is necessary for ligand binding [91]. Altered glycosylation of EGFR has been shown in colorectal cancer samples compared to adjacent normal tissue [92]. In addition, statin treatment altered glycosylation of surface proteins in liver cells [93].

Therefore, prenylation inhibition by statins and N-bisphosphonates are expected to alter EGFR signaling by disrupting lipid raft localization and inhibiting n-glycosylation, further complicating evaluation of efficacy on Ras, more specifically on KRAS localization. However, FTis inhibitory range—specifically inhibiting farnesylation—are much narrower warranting combination studies along with mevalonate pathway inhibitors to more specific investigation of KRAS inhibition.

## Concluding remarks

Despite promising feasibility of prenylation inhibition of KRAS mutant tumors, successful clinical application of this concept has been hindered by several factors, some of them discussed in this review. First of all, there is a lack of prospective, randomized clinical trials specifically addressing whether prenylation inhibitors like statins; N-bisphosphonates have any direct antitumor effect on KRAS mutant solid tumors. Notably, trials using FTis and statins had many handicaps like involving advanced metastatic tumors that are very difficult to target or utilizing low doses that are also not expected to reach high enough concentration for antitumoral effects (Table 1).

Furthermore, recently developed lipophilic N-bisphosphonates are also potential applicants for targeting even non-bone-related KRAS mutant tumors [48, 71]. N-bisphosphonates may have better toxicity profile and lower drug-to-drug interaction making them promising candidates for combinational therapy [43, 44, 49].

Next, it is urgently needed to develop and utilize biomarkers for KRAS dependency so that patients with potential benefits for KRAS targeting can be successfully identified. Using Ras signature may be a possible option [94], and indeed, one clinical trial with statin plus cetuximab/irinotecan treatment found association of response with Ras signature (Table 1). Of note, development of this score showed that mutation in KRAS is not a prerequisite for KRAS dependency [94].

Last but not least, our basic understanding of KRAS biology still has many gaps to cover. One major lack of knowledge is related to the dynamics of K-Ras protein turnover. Dynamics of K-Ras synthesis and degradation is still unclear and these processes are crucial for prenylation inhibition as this approach can only target newly synthesized K-Ras proteins.

## Data Availability

Upon reasonable request data used for the presented analysis is available from the corresponding author.
